# Severe hypereosinophilia secondary to intestinal whipworm infection with negative stool tests: a case report

**DOI:** 10.3389/fmed.2026.1872657

**Published:** 2026-06-22

**Authors:** Jia Chen, Yueying Chen, Fu Su

**Affiliations:** Department of Gastroenterology, Air Force Hospital of PLA Southern Theater Command, Guangzhou, Guangdong, China

**Keywords:** albendazole, case report, colonoscopy, diarrhea, eosinophilia, whipworm infection

## Abstract

**Background:**

Severe hypereosinophilia is commonly associated with hematologic disorders, whereas parasitic infections are often underrecognized, particularly in non-endemic settings. The diagnosis can be further complicated when routine stool examinations are negative.

**Case presentation:**

A 24-year-old man presented with a 2-month history of chronic diarrhea and marked hypereosinophilia (11.56 × 10^9^/L). Initial investigations, including repeated stool examinations and colonoscopy, failed to identify an underlying cause, leading to diagnostic uncertainty. The patient was initially treated for a nonspecific gastrointestinal disorder, with only transient symptomatic relief. Upon readmission, a repeat colonoscopy revealed two Trichuris trichiura worms in the cecum and ascending colon, which were successfully removed endoscopically. Histopathological examination demonstrated prominent eosinophilic infiltration. Following a short course of albendazole (400 mg once daily for 3 days), the patient’s symptoms resolved completely, and eosinophil counts rapidly normalized, with no residual gastrointestinal symptoms during follow-up.

**Conclusion:**

Clinicians should maintain a high index of suspicion for parasitic infections in patients with unexplained hypereosinophilia and gastrointestinal symptoms. Repeated evaluations, including colonoscopy, may be essential for establishing the diagnosis and guiding timely treatment.

## Introduction

Whipworm (Trichuris trichiura) is a soil-transmitted nematode that primarily inhabits the human large intestine, particularly the cecum and ascending colon, with humans serving as its definitive host. The parasite embeds its slender anterior end into the intestinal mucosa and feeds on host tissue fluid and blood, causing local mucosal injury and triggering host immune responses (1). Clinically, T. trichiura infection may be asymptomatic or present with nonspecific gastrointestinal manifestations, including anorexia, abdominal pain, chronic diarrhea, weight loss, hematochezia, and, in heavier infections, anemia or systemic allergic symptoms (1, 2). From an epidemiological perspective, T. trichiura remains an important parasitic infection worldwide. Although its prevalence has declined in many regions because of improved sanitation, public health measures, and living conditions, the disease has not disappeared. It continues to be endemic in areas with inadequate sanitation, particularly in parts of sub-Saharan Africa, Southeast Asia, and Latin America (3, 4). Sporadic cases may also occur in non-endemic regions, including imported infections, infections in travelers, and overlooked infections in vulnerable populations. This changing epidemiological pattern has created a clinical paradox: as parasitic infections become less common in daily practice, clinicians may become less likely to consider them (5).

Eosinophilia is an important immunological clue in helminth infection. It is generally defined as an absolute peripheral blood eosinophil count greater than 500/μL, whereas severe eosinophilia is usually diagnosed when the count reaches or exceeds 5.0 × 10^9^/L (6). In parasitic infections, eosinophil expansion is largely driven by type 2 immune responses. Parasite invasion and parasite-related allergic reactions activate Th2 pathways and promote the release of cytokines such as IL-4, IL-5, and IL-13, which in turn mediate IgE production, mast-cell activation, eosinophil proliferation, and eosinophil release into the systemic circulation (7, 8). In practice, however, severe eosinophilia rarely points to a single diagnosis. Hematologic disorders, allergic diseases, autoimmune conditions, drug reactions, and idiopathic hypereosinophilic syndromes are often considered early in the diagnostic process (9). Whipworm infection may be less readily suspected, particularly in areas where soil-transmitted helminthiasis is now uncommon. The problem is compounded by its nonspecific gastrointestinal presentation, which can resemble irritable bowel syndrome, inflammatory bowel disease, eosinophilic gastroenteritis, or other common intestinal disorders. Moreover, stool examination may be negative when worm burden is low, egg shedding is intermittent, or sampling is limited; endoscopy may also miss parasites located near the ileocecal region or associated with only subtle mucosal changes (3–5, 10).

Although T. trichiura infection has been reported in patients with eosinophilia, most published cases describe mild to moderate eosinophil elevation. Severe hypereosinophilia meeting established diagnostic thresholds remains poorly characterized (11). This gap is clinically relevant because negative stool tests or an unrevealing initial colonoscopy may lead clinicians away from a parasitic cause. The present case is therefore not simply a report of whipworm infection, but an example of how an occult intestinal parasite can mimic more common causes of severe hypereosinophilia. The patient had chronic diarrhea, marked eosinophilia, repeatedly negative stool examinations, and an initially unremarkable colonoscopy. The diagnosis was established only after repeat colonoscopy identified T. trichiura in the cecum and ascending colon, and the causal relationship was supported by rapid clinical improvement and normalization of eosinophil counts after albendazole therapy. By describing this diagnostic course, we aim to emphasize that intestinal parasitic infection should remain in the differential diagnosis of unexplained severe hypereosinophilia, even when initial stool and endoscopic examinations are negative.

## Case presentation

A 24-year-old man was admitted to the Department of Gastroenterology, Southern Theater Command Air Force Hospital, on September 9, 2025, because of persistent diarrhea for 2 months. In early July 2025, he developed watery diarrhea without an obvious trigger, occurring 5–10 times per day. The stools were yellowish-green and were accompanied by intermittent mid-lower abdominal pain. He initially underwent colonoscopy at an outside hospital, which showed no obvious abnormality throughout the colon. Oral berberine hydrochloride was prescribed, but his symptoms did not improve.

In late August, the clinical course became more complicated. The patient developed fever together with swelling and pain of the right lower limb. After 7 days of anti-infective therapy, the fever subsided, the limb swelling improved, and the diarrhea also temporarily eased. However, the gastrointestinal symptoms recurred shortly after treatment was discontinued. He was therefore referred to our hospital for further evaluation of diarrhea of unknown origin. He denied any recent travel history to parasite-endemic areas. He denied hypertension, diabetes mellitus, coronary heart disease, previous surgery, trauma, smoking, alcohol use, or exposure to special chemicals.

### Physical examination

On admission, his vital signs were stable, with a temperature of 36.2 °C, pulse rate of 78 beats/min, respiratory rate of 20 breaths/min, and blood pressure of 118/80 mmHg. He was alert and oriented, with a mildly anemic appearance. No jaundice was observed. His skin is good in elasticity and normal in moisture, with no signs of dehydration Cardiopulmonary examination was unremarkable. The abdomen was soft and flat, without tenderness, rebound tenderness, palpable mass, hepatosplenomegaly, or abnormal bowel sounds.

### Laboratory and imaging examinations

Initial laboratory testing revealed marked leukocytosis with striking eosinophilia. The white blood cell count was 16.5 × 10^9^/L, with eosinophils accounting for 70.1% of leukocytes and an absolute eosinophil count of 11.56 × 10^9^/L. Hemoglobin was 129 g/L, and the platelet count was 256 × 10^9^/L. Stool routine examinations were performed on three separate specimens. Microscopic examination revealed white blood cell counts of 0–3 per high-power field in one specimen and 0–2 per high-power field in the other two. Fecal occult blood test yielded weakly positive results in all samples, while no parasite ova were identified in any specimen. Stool specimens were collected on September 10, 12 and 14, 2025. All samples were examined by a qualified parasitological technician via direct smear and saline floatation concentration techniques. No Salmonella and Shigella was detected in the stool culture was performed. Serum total IgE was measured and found to be elevated at 680 IU/mL (reference range < 100 IU/mL). Liver and renal function tests, electrolytes, and inflammatory markers were essentially within normal ranges. Abdominal ultrasonography showed no obvious abnormalities in the liver, gallbladder, pancreas, spleen, or kidneys. Gastroscopy revealed chronic non-atrophic gastritis. Peripheral blood smear was performed and showed mature eosinophilia without abnormal lymphocytes or blasts. Bone marrow assessment and molecular assays for clonal eosinophilia, including FIP1L1-PDGFRA rearrangement detection, were omitted. This decision was made considering the invasiveness of bone marrow puncture, as colonoscopy reexamination confirmed parasitic etiology and obvious clinical improvement was achieved after anthelmintic treatment. Systemic autoimmune screening (ANA, ANCA and other indexes) were essentially within normal ranges. Targeted allergy tests were not implemented, with only a prior history of allergic dermatitis documented. Strongyloides stercoralis and other helminth infections were not specifically investigated by serological testing or special stool examinations (such as Baermann funnel method). Eosinophilic gastroenteritis was considered in the differential diagnosis; however, the identification of whipworms during colonoscopy and the dramatic clinical response to parasite removal made eosinophilic gastroenteritis unlikely. These diagnostic limitations should be acknowledged.

At this stage, the combination of chronic diarrhea and severe hypereosinophilia raised concern for several possible diagnoses, including eosinophilic gastroenteritis, inflammatory bowel disease, allergic disease, parasitic infection, and hematologic disorders. However, the absence of parasite eggs in stool and the previously unremarkable colonoscopy made the diagnosis uncertain.

### Treatment and clinical outcome

**Figure 1:**
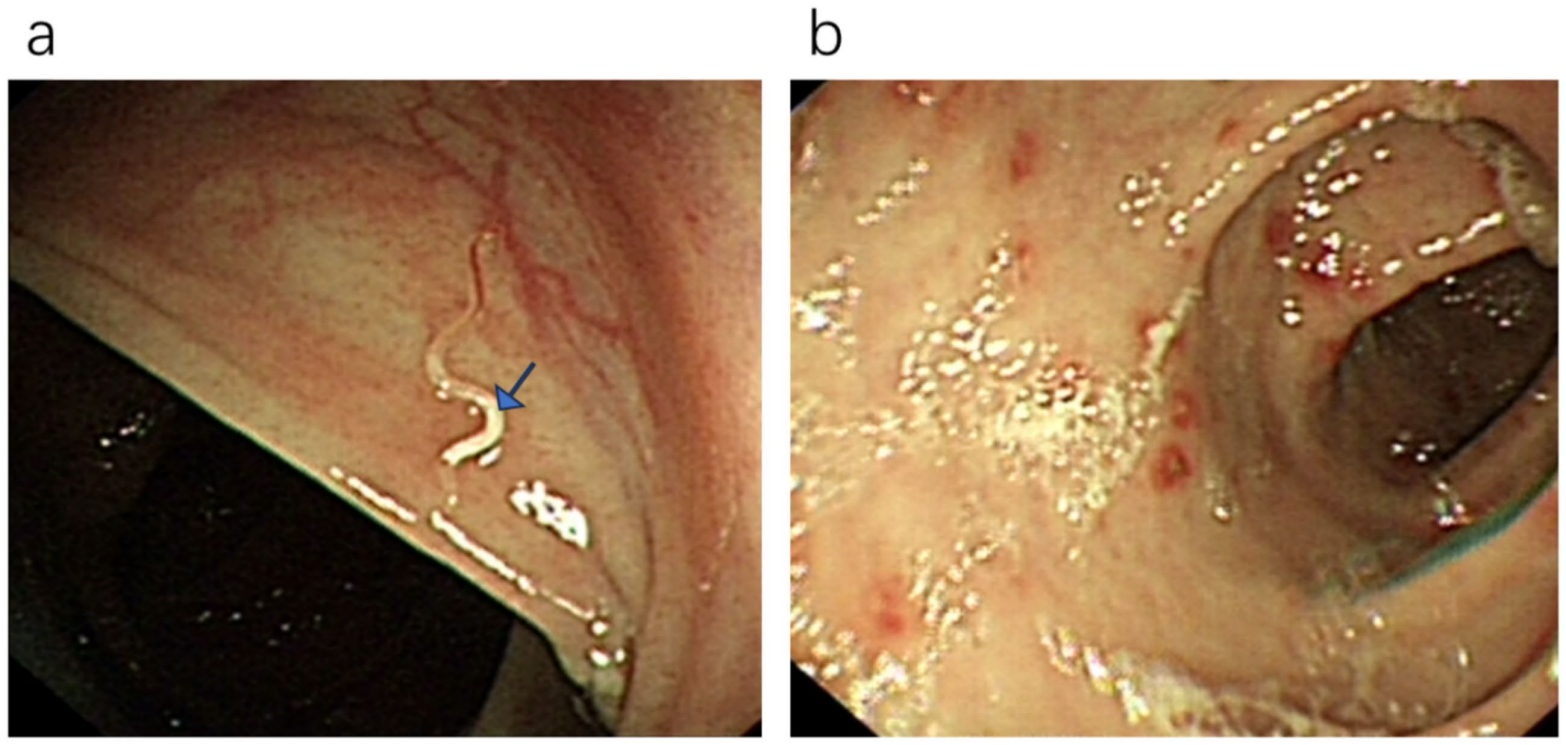
Colonoscopic findings: Whipworms (arrows) identified in the cecum andascending colon near the ileocecal valve **(a)**, with scattered mucosal hyperemia anderosion in the surrounding area **(b)**.

**Figure 2:**
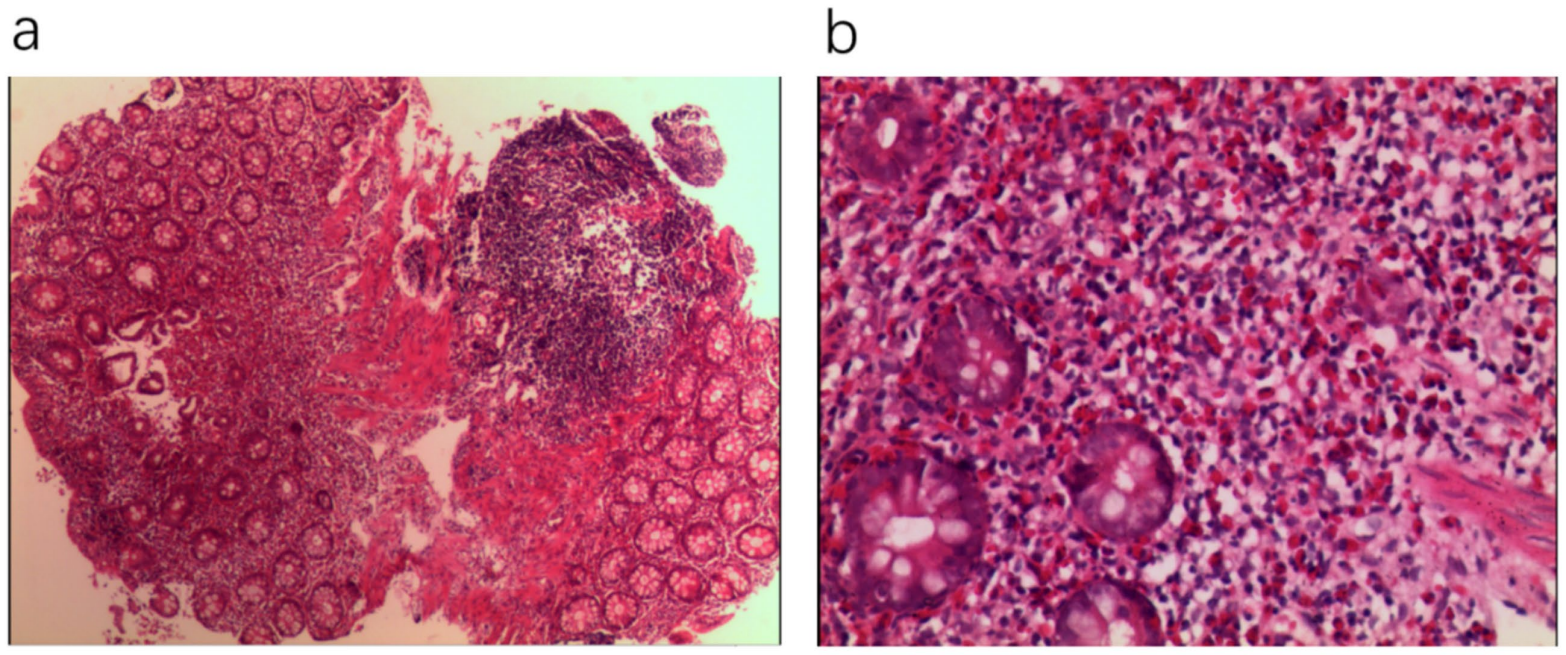
Pathological findings (HE staining): **(a)** Diffuse infiltration of a large number of eosinophils in the intestinal mucosa of the ileocecal region and ascending colon (×200); **(b)** Magnified view showing eosinophilic infiltrates predominantly distributed in the lamina propria, with eosinophils also present in the superficial submucosa (×400).

After admission, the patient received symptomatic treatment, including oral *Bifidobacterium infantis* powder, montmorillonite powder, and a liquid diet. Despite these measures, diarrhea and abdominal pain persisted. Given the unexplained severe eosinophilia and ongoing gastrointestinal symptoms, colonoscopy was repeated on September 17. This time, two slender whitish worms were identified in the cecum and ascending colon near the ileocecal valve (Figure 1). The worms were approximately 3.5–4.0 cm in total length, with a characteristic thin, whiplike anterior portion (approximately 3/5 of body length) and a thicker posterior portion. The worms were alive and motile during endoscopy. The surrounding mucosa showed scattered hyperemia and erosion. The worms were removed endoscopically using biopsy forceps. Based on their morphology and location, intestinal whipworm infection was suspected. Histopathological examination of the adjacent intestinal mucosa demonstrated prominent eosinophilic infiltration, further supporting parasite-associated mucosal inflammation (Figure 2). The eosinophilic infiltrates were predominantly distributed in the lamina propria of the ileocecal region and ascending colon, with eosinophils also present in the superficial submucosa. Cryptitis was not observed. No granulomas, ulceration, or eosinophilic microabscesses were identified in the biopsy specimens. Parasite structures were not observed within the intestinal tissue.

On September 18, the patient was treated with oral albendazole 400 mg once daily for 3 consecutive days. His diarrhea gradually improved, and abdominal pain resolved after treatment. A repeat blood count on September 21 showed a marked decline in eosinophilia: the white blood cell count decreased to 8.96 × 10^9^/L, eosinophils accounted for 18.2%, and the absolute eosinophil count fell to 5.13 × 10^9^/L. The patient remained clinically stable and was discharged. This clinical improvement demonstrates the direct causal relationship between parasite removal and symptom resolution.

### Follow-up

**Figure 3:**
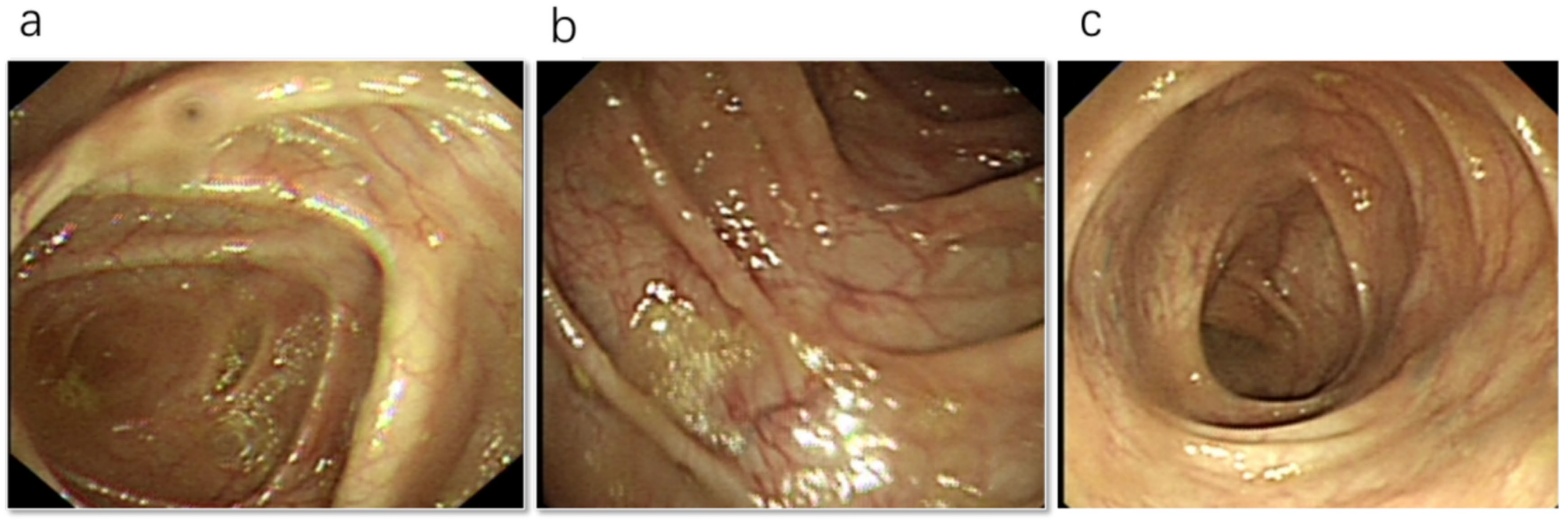
Colonoscopic findings at follow-up: **(a)** Normal colonic mucosa in the cecum, with no hyperemia or erosion; **(b)** Normal ascending colon mucosa with clear vascular pattern; **(c)** Normal transverse colon mucosa with distinct circular folds.

**Figure 4:**
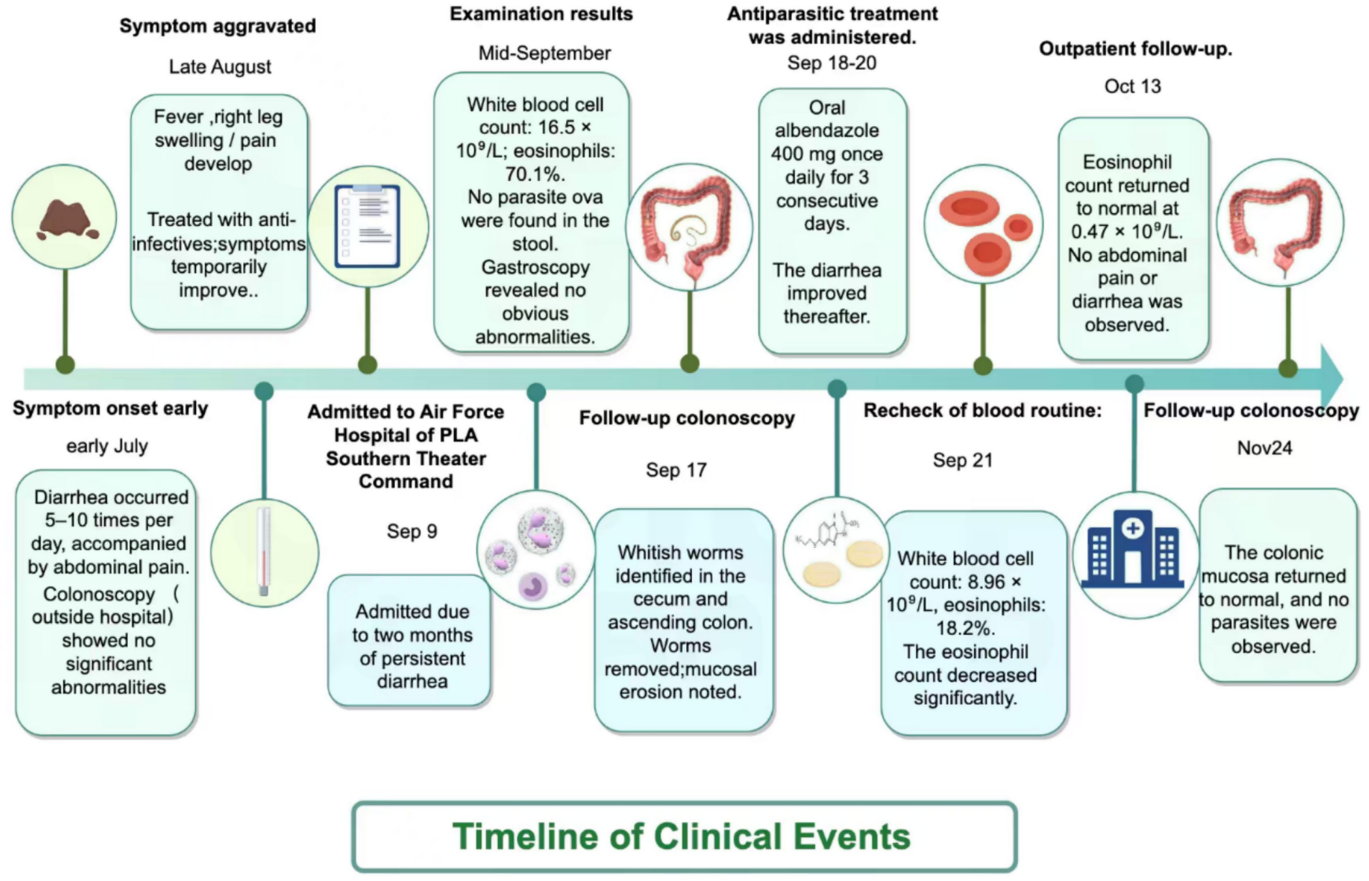
The complete diagnostic and therapeutic timeline for this patient.

During follow-up, the clinical and laboratory response further supported the causal relationship between whipworm infection and severe eosinophilia. On October 13, peripheral blood testing showed a white blood cell count of 3.98 × 10^9^/L, eosinophils of 11.8%, and an absolute eosinophil count of 0.47 × 10^9^/L, which had returned to the normal range. The patient reported no recurrent diarrhea, abdominal pain, or other gastrointestinal discomfort. Follow-up colonoscopy on November 24 showed normal colonic mucosa, with no residual worms, erosions, or other mucosal lesions (Figure 3). Figure 4 shows the complete diagnostic and therapeutic timeline for this patient.

## Discussion

The most striking feature of this case was the discrepancy between the severity of eosinophilia and the apparently limited parasitic burden. The patient had an absolute eosinophil count of 11.56 × 10^9^/L, yet only two adult whipworms were identified during colonoscopy. At first glance, such a small number of visible parasites might seem insufficient to explain the degree of eosinophilia. However, the clinical course argues otherwise. The worms were found at the site typically involved in T. trichiura infection, the adjacent mucosa showed prominent eosinophilic infiltration, and both symptoms and eosinophil counts improved rapidly after endoscopic removal and albendazole therapy (12, 13). The case therefore illustrates a practical clinical problem: in patients with severe eosinophilia and gastrointestinal symptoms, a negative initial evaluation does not necessarily exclude an intestinal parasite (14, 15). In a patient with chronic diarrhea and eosinophilic infiltration of the intestinal mucosa, eosinophilic gastroenteritis is an understandable consideration. Hematologic disorders and hypereosinophilic syndromes may also come to mind when the eosinophil count is markedly elevated. The difficulty is that these diagnoses are often considered before infection has been fully excluded. In the present case, the intestinal eosinophilia was more likely a secondary reaction to mucosal parasitic invasion than a primary eosinophilic gastrointestinal disorder. This distinction matters because the therapeutic implications are completely different (16, 17). Notably, severe hypereosinophilia (absolute eosinophil count ≥5.0 × 10^9^/L) is uncommon in Trichuris trichiura infection. Previous case reports have documented eosinophil levels ranging from mild elevation (800–1,500/μL) to moderate elevation, with severe hypereosinophilia being exceedingly rare. In the present case, the peak absolute eosinophil count reached 11.56 × 10^9^/L, which is disproportionately severe relative to the low visible worm burden (only two worms identified). The possible immunological mechanisms explaining this disproportionate eosinophilic response include: (1) Host factors—the patient had a history of allergic dermatitis, suggesting a Th2-skewed immune background that may amplify eosinophil production upon parasite antigen exposure; (2) Antigen load from living, motile worms in the cecum may have continuously stimulated a robust Th2 response, with elevated IgE (680 IU/mL) supporting this mechanism; (3) Occult additional worm burden cannot be excluded—microscopic eggs may be missed in stool examinations, and imaging studies have shown that up to 40% of symptomatic whipworm infections may have low egg counts or be egg-negative. Therefore, the severe eosinophilia in this case may reflect a combination of true low worm burden with host immune hyper-responsiveness.

The negative stool examinations were central to the delay in diagnosis. Stool microscopy is useful, but it is not definitive in every case. Egg detection depends on several practical factors, including worm burden, egg output, timing of sampling, specimen quality, and laboratory technique (15). A patient harboring only a few worms may have a low or intermittent egg burden in stool, and repeated negative results can give false reassurance. This case therefore cautions against treating negative stool tests as conclusive when the clinical picture remains unexplained. Whipworms are small, thin, and often attached to the mucosa of the cecum or proximal colon. When only a few worms are present, they may be hidden by folds, mucus, or residual intestinal contents, particularly around the ileocecal region. The fact that repeat colonoscopy revealed two worms in the cecum and ascending colon suggests that the first examination may simply have missed a subtle lesion rather than ruling out intestinal parasitosis. For patients with persistent gastrointestinal symptoms and marked eosinophilia, careful inspection of the cecum, ascending colon, and ileocecal valve is therefore important (12, 13).

Another point worth emphasizing is the value of treatment response. In case reports, causality is often difficult to prove. Here, however, the sequence was persuasive: the parasite was visualized and removed, albendazole was administered, gastrointestinal symptoms resolved, and the eosinophil count fell quickly before returning to the normal range during follow-up. This temporal relationship, together with the pathological finding of eosinophilic infiltration, makes whipworm infection the most coherent explanation for the clinical presentation.

### Strengths and limitations of this case report

*Strengths*: This case report provides detailed endoscopic and pathological findings, which are essential for the diagnosis of intestinal whipworm infection. The follow-up data, including peripheral blood eosinophil counts at multiple time points and repeat colonoscopy results, demonstrate the complete clinical course from diagnosis to treatment and recovery. These detailed records strengthen the causal relationship between parasite removal and clinical improvement.

*Limitations*: This is a single case report, which limits the generalizability of the findings. The diagnosis was confirmed by colonoscopy rather than the gold-standard stool egg detection, which is a common scenario in clinical practice when stool examinations are negative. Additionally, long-term follow-up data beyond November 2025 are not available, and the patient’s immunological workup was not comprehensive enough to fully exclude other causes of hypereosinophilia. Specifically, serum IgE was measured and found elevated, but peripheral blood smear, bone marrow study, and molecular testing for clonal eosinophilia were not performed. Autoimmune and systematic allergic evaluations were not conducted. Strongyloides stercoralis and other helminths were not specifically investigated. Eosinophilic gastroenteritis was considered but not formally excluded by full-thickness bowel biopsy. These diagnostic limitations should be acknowledged, as they may affect the completeness of the differential diagnosis.

## Conclusion

In conclusion, severe eosinophilia should not automatically lead clinicians toward complex hematologic or immune-mediated diagnoses, particularly when gastrointestinal symptoms are present. Those diagnoses may still need to be considered, but treatable secondary causes should be actively sought first. Similarly, eosinophilic infiltration on intestinal biopsy should not be interpreted in isolation. Without careful exclusion of infection, it may lead to an erroneous diagnosis of primary eosinophilic gastroenteritis. This case demonstrates that occult T. trichiura infection may present with severe hypereosinophilia, even when stool examinations and initial colonoscopy are unrevealing. When marked eosinophilia remains unexplained, especially in a patient with chronic diarrhea or abdominal pain, repeat evaluation for intestinal parasites is justified. Careful colonoscopic inspection of the proximal colon may be decisive.

## Data Availability

The original contributions presented in the study are included in the article/supplementary material, further inquiries can be directed to the corresponding author.
